# Genetic confirmation of a hybrid between two highly divergent cardinalid species: A rose‐breasted grosbeak (*Pheucticus ludovicianus*) and a scarlet tanager (*Piranga olivacea*)

**DOI:** 10.1002/ece3.9152

**Published:** 2022-08-01

**Authors:** David P. L. Toews, Tessa A. Rhinehart, Robert Mulvihill, Spencer Galen, Stephen M. Gosser, Tom Johnson, Jessie L. Williamson, Andrew W. Wood, Steven C. Latta

**Affiliations:** ^1^ Department of Biology, 619 Mueller Laboratory Pennsylvania State University University Park Pennsylvania USA; ^2^ Department of Biological Sciences University of Pittsburgh Pittsburgh Pennsylvania USA; ^3^ Department of Conservation and Field Research National Aviary Pittsburgh Pennsylvania USA; ^4^ Biology Department University of Scranton Scranton Pennsylvania USA; ^5^ Pittsburgh Pennsylvania USA; ^6^ The Academy of Natural Sciences of Drexel University Philadelphia Pennsylvania USA; ^7^ Department of Biology and Museum of Southwestern Biology University of New Mexico Albuquerque New Mexico USA

**Keywords:** birds, genomics, hybridization, reproductive isolation

## Abstract

Using low‐coverage whole‐genome sequencing, analysis of vocalizations, and inferences from natural history, we document a first‐generation hybrid between a rose‐breasted grosbeak (*Pheucticus ludovicianus*) and a scarlet tanager (*Piranga olivacea*). These two species occur sympatrically throughout much of eastern North America, although were not previously known to interbreed. Following the field identification of a putative hybrid, we use genetic and bioacoustic data to show that a rose‐breasted grosbeak was the maternal parent and a scarlet tanager was the paternal parent of the hybrid, whose song was similar to the latter species. These two species diverged >10 million years ago, and thus it is surprising to find a hybrid formed under natural conditions in the wild. Notably, the hybrid has an exceptionally heterozygous genome, with a conservative estimate of a heterozygous base every 100 bp. The observation that this hybrid of such highly divergent parental taxa has survived until adulthood serves as another example of the capacity for hybrid birds to survive with an exceptionally divergent genomic composition.

## Graphical abstract

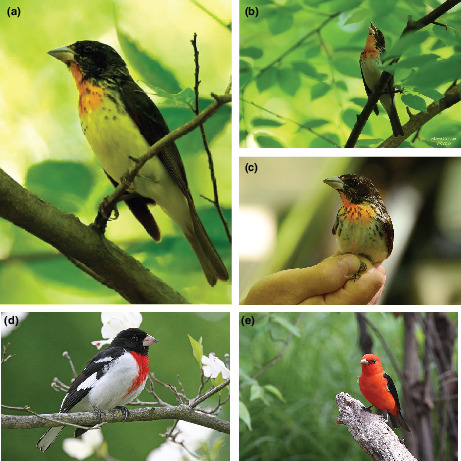

## 1 INTRODUCTION

Identifying the putative parental species of naturally occurring hybrid birds is becoming more feasible with the breadth of genomic data becoming available (Talbot et al., 2011; Toews et al., 2020; Toews, Streby, et al., 2018). This has been paralleled by significant developments in machine learning methods to identify species from photographs or sound recordings (Wäldchen & Mäder, 2018). Determining hybrid ancestry sometimes begins as a curiosity‐driven pursuit (Parkes, 1978); however, given the growing number of divergent hybrids confirmed with molecular tools, several authors have begun making broader useful generalizations on the evolution of reproductive isolation (e.g., Rothfels et al., 2015). Moreover, in avian systems, research on the coloration patterns observed in hybrids between divergent parents has been used to learn about the inheritance of plumage and song traits (Williamson et al., 2021). Hybridization among bird species, in particular, is known to be common (Grant & Grant, 1992), yet the majority of this occurs between very closely related species and within hybrid zones.

Here, we apply genomic and bioacoustic analyses to document the first described hybrid between two highly divergent species, rose‐breasted grosbeak (*Pheucticus ludovicianus*) and scarlet tanager (*Piranga olivacea*), which occur sympatrically throughout much of eastern North America. Both species are members of the Cardinalidae family and have not previously been known to hybridize. Moreover, based on the time‐calibrated phylogeny of Barker et al. (2015), they last shared a common ancestor >10 million years ago. While postzygotic incompatibilities have been shown to take much longer in birds (Fitzpatrick, 2004), overall reproductive isolation is generally thought to be complete after approximately 2–4 million years in high‐latitude avian species pairs where this has been studied (Price, 2008; Weir et al., 2015), making this naturally occurring, wild hybrid unusual. We use genomic data, combined with song recording, to confirm field assessment of the parental species, as well as quantify genome‐wide patterns of heterozygosity.

## 2 METHODS

### 2.1 Observation

**FIGURE 1:**
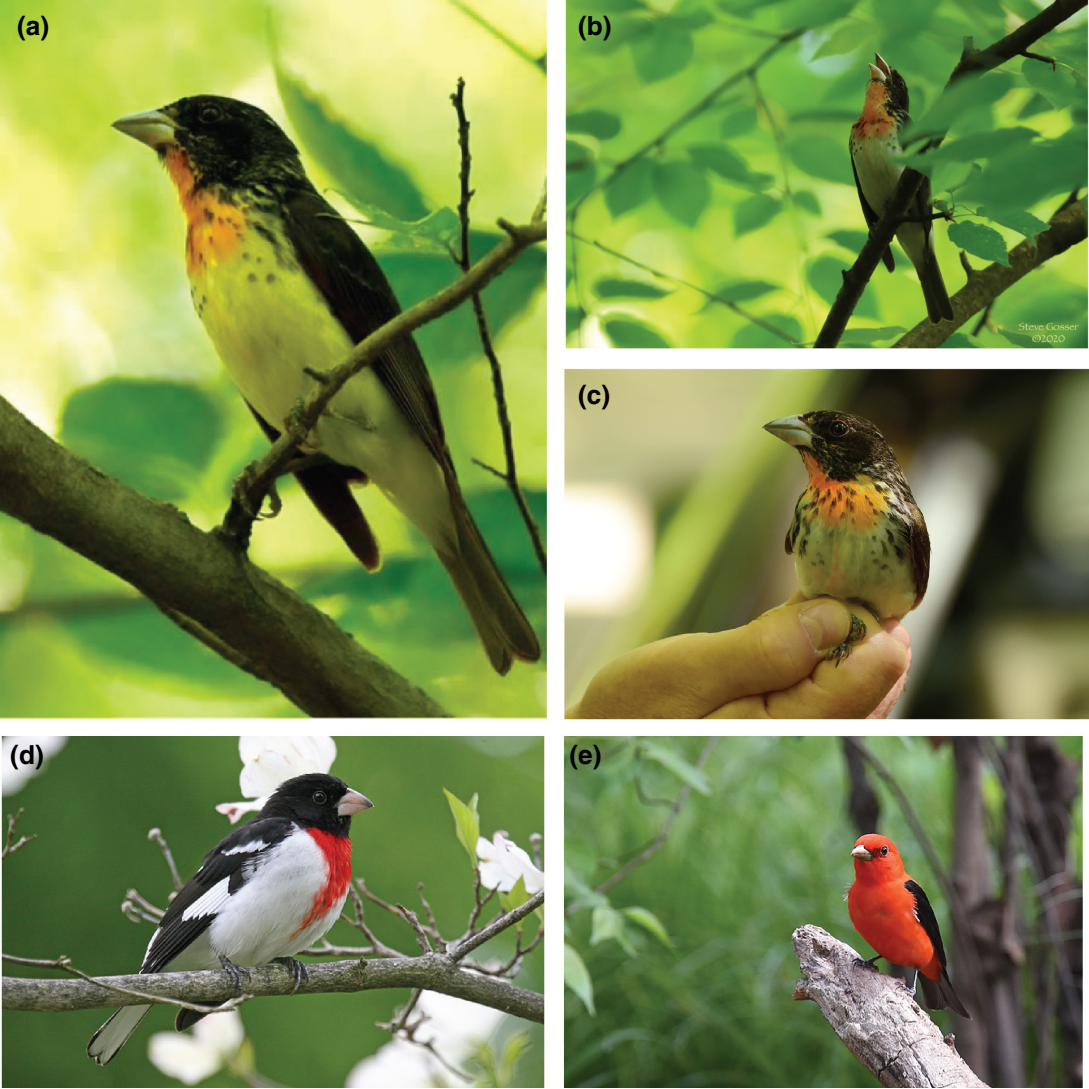
Photographs of the hybrid from the front (a), while singing (b), and in the hand (c). The putative parental species: A rose‐breasted grosbeak (d) and a scarlet tanager (e). Photos A–C by Steve grosser, D by John Harrison (cc‐by‐2.0), and E by Andy Reago & Chrissy McClarren (cc‐by‐2.0).

On June 6, 2020, in Lawrence County, Pennsylvania, S.M.G. heard a song that he took to be a scarlet tanager. He searched for the bird in order to take a photograph, which instead looked like male rose‐breasted grosbeak but with marked differences in plumage and morphology (Figure 1). On June 7, 2020, R.M. successfully re‐located the singing bird and mist netted it using an audio lure of tanager song. Plumage differences distinguishing the individual from a typical male rose‐breasted grosbeak included its black wings and tail without white markings, yellowish white underwings instead of pink, and a pink instead of black throat. It also had a small concealed pale yellow crown patch (Figure 1a‐d). Morphological differences included a longer primary projection and a more elongated, shallower bill that was darker and more gray‐green than the pink‐ivory bill of a rose‐breasted grosbeak. Its bill lacked a tomial tooth, a characteristic of *Piranga* tanagers. S.L. then extracted 5–10 μl of blood by venipuncture from the ulnar vein in the wing, which was then stored on Whatman™ filter paper. R.M. and S.L. then collected standard morphological measurements of the bird (Table 1). Bird handling was approved by the Institutional Animal Care and Use Committee of the National Aviary and Pittsburgh Zoo and PPG Aquarium.

**TABLE 1:** Comparative morphological measurements of male scarlet tanager (SCTA) male rose‐breasted grosbeak (RBGR), and a hybrid second‐year male of these species captured in June.

Species	Mass	Wing chord	Tail	Tarsus	Exposed culmen	Nares to tip	Depth at nares
SCTA	25.0 (23.5–33.0)^c^	94.5 ± 2.5^a^	63–72^b^	19.8	10.4 + 0.6 SD^a^	10.5–12.1^c^	7.3–8.2^b^
RBGR	43.6 (36.9–52.5)^d^	100.2 ± 2.9^b^	74.6 (70.1–78.5)^e^	22.5 (22.0–24.0)^e^	16.9 (15.4–18.2)^e^	12.3^f^	13.2^g^
SCTA × RBGR	35.0	97.5	79.5	20.5	17.0	12.5	10.0

^a^ Holmes, 1986.

^b^ Pyle, 1997.

^c^ Mowbray, 2020.

^d^ Clench & Leberman, 1978.

^e^ Godfrey, 1986.

^f^ Kroodsma, 1986.

^g^ Ricklefs, 2017.

### 2.2 Bioacoustic analysis

T.J. audio‐recorded vocalizations of the individual between 5:40 and 8:00 a.m. on the morning of June 11, 2020, using a Wildtronics Pro Mono microphone mounted in a 22” Wildtronics parabolic reflector and a Sound Devices MixPre‐3 digital audio recorder. Files were recorded as lossless. WAV files, and lightly edited using the iZotope RX 6 audio editor. The three recordings of the bird totaled approximately 5.5 min of recording of song and calls. Two of these recordings contained the putative hybrid singing alone (*n* = 25 songs). The third recording captured a counter‐singing interaction between the putative hybrid and a scarlet tanager. Recordings have been deposited in the Macaulay Library of Natural Sounds (ML462228091, ML462228311, ML462228571, ML462231831).

**FIGURE 2:**
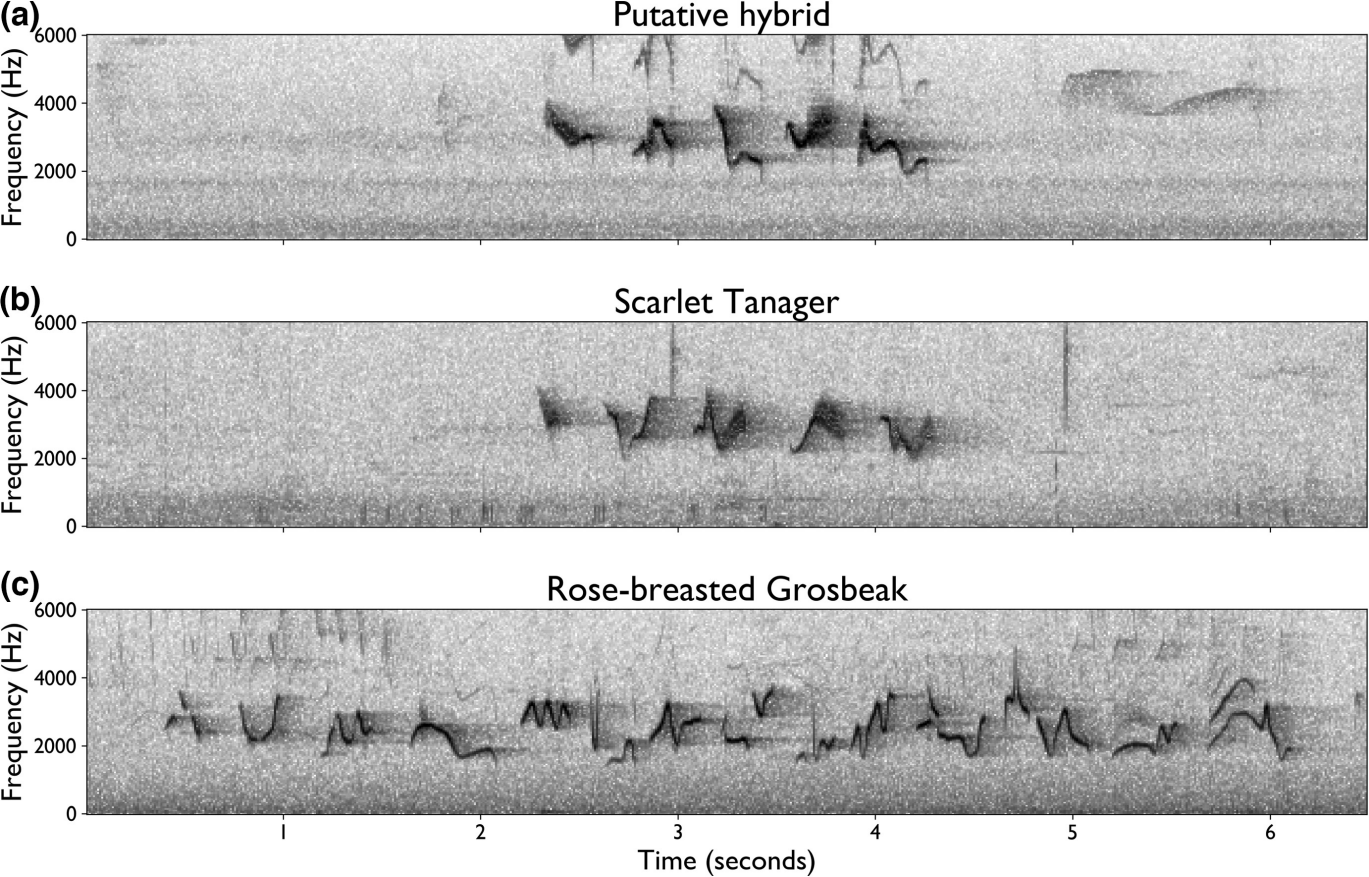
Spectrograms of (a) hybrid Scarlet Tanager x Rose‐Breasted Grosbeak song, (b) scarlet tanager song (Jim Berry, XC317656) and (c) rose‐breasted grosbeak song (Jim Berry, XC372244).

We used Raven Pro Sound Analysis Software v1.5 (K. Lisa Yang Center for Conservation Bioacoustics, 2014) to assess the characteristics of the *n* = 25 of recorded songs, excluding the two songs captured in the counter‐singing interaction. For each recording, we generated a spectrogram, a visual representation of the sound with time on the horizontal axis, frequency on the vertical axis, and amplitude (or “loudness”) represented by the darkness of the pixel (Figure 2). We used the annotation feature of Raven Pro to identify the time and frequency boundaries of the syllables within the recorded songs. We then compared measurements of the songs' frequency range, number of syllables, and duration of the putative hybrid's song to those of previously published measurements of those attributes in scarlet tanager and rose‐breasted grosbeak songs (Table 2; Mowbray, 2020; Wyatt & Francis, 2020). While we did not include the songs in the counter‐singing recording in this assessment, we did annotate which songs belonged to which individual. Annotation files are available in Data Dryad Repository https://doi.org/10.5061/dryad.wm37pvmqs.

**TABLE 2:** Comparison of vocalizations of putative hybrid to those of scarlet tanager (SCTA) and rose‐breasted grosbeak (RBGR).

Characteristic	Hybrid (*n* = 25 songs)	SCTA (Mowbray, 2020)	RBGR (Wyatt & Francis, 2020)
Rapid frequency modulation	yes	yes	no
Frequency range	1.2–5.9 kHz (full range)	2.2–5.5 kHz (typical range)	1.5–5 kHz (typical range)
No. syllables per song	4–6 (4.96 ± 0.68)	1–7, most often 4–5	3–20 or more; most often 10
Song duration	1.28–2.30 s (1.79 s ± 0.25)	1.5–4.0 s	3–5 s
“Chick‐burr” call	yes	yes	no

*Notes*: Values for the putative hybrid are shown with mean ± SD. Vocalization information from SCTA and RBGR summarized from Birds of the World species accounts (Mowbray, 2020; Wyatt & Francis, 2020).

### 2.3 Genomic methods

To estimate the genetic ancestry of the putative hybrid, we used low‐coverage whole‐genome sequencing as in Toews et al. (2020). We first extracted DNA from the blood sample obtained from the putative hybrid, using Qiagen DNAeasy spin columns and following manufacturer protocol (Qiagen). We then generated short‐read genomic data from the hybrid using an Illumina TruSeq Nano library preparation kit (Illumina) targeting a 350 bp insert size. The hybrid sample was included within a larger sequencing project focused on parulid warblers. For sequencing, 24 individuals were individually indexed and pooled on an Illumina NextSeq 500 lane using paired‐end 150 bp sequencing chemistry (Penn State Genomics Core Facility). The hybrid sample was bioinformatically de‐multiplexed from the other individuals included in the lane not related to the present study. The data for the hybrid is deposited in the NCBI SRA (Bioproject #PRJNA861242).

We compared read data of the hybrid with previously published short‐read sequence data deposited in the NCBI Short Read Archive (SRA) to confirm the maternal parent species and to identify the putative paternal parent. The comparison species for the putative parents of the hybrid (a rose‐breasted grosbeak, *Pheucticus ludovicianus*, and a scarlet tanager, *Piranga olivacea*) were chosen based on preliminary mitochondrial DNA (mtDNA) sequencing, morphological similarity, and qualitative song characteristics. The closest available complementary short‐read datasets that included both parental *genera* (as of June 2021) were derived from an RNA‐seq study of blood investigating hemosporidian parasites (Galen et al., 2020). This included the rose‐breasted grosbeak (*Pheucticus ludovicianus*; SRA Accession #SAMN11263484) and the western tanager (*Piranga ludoviciana*; SRA Accession #SAMN11263491; MSB:Bird:47847), but not the specific putative paternal parent species, the scarlet tanager (*Piranga olivacea*). Western tanagers do not occur in the eastern USA where the hybrid was reported, and beyond a scarlet tanager, the only other member of the *Piranga* genus that occurs in the region where the hybrid was reported is *Piranga rubra* (summer tanager). However, we were able to use genetic data to identify genus‐level assignment for the paternal parent and use other inferences to assign species‐level identity (see below).

We used AdapterRemoval (Lindgreen, 2012) to collapse overlapping read pairs and trim low‐quality bases from read ends. We aligned these reads to the only high‐quality cardinalid genome publicly available, from the northern cardinal (*Cardinalis cardinalis*; GenBank assembly accession # GCA_014549065.1; Sin et al., 2020) using BowTie2 (Langmead & Salzberg, 2012). We added to this assembly the full mitochondrial genome sequence from a separate *C. cardinalis* individual (NCBI GenBank accession #MH700631) to facilitate the alignment of mtDNA reads. For the data from the putative hybrid, we used read‐pair information and set the maximum distance between pairs (the ‐X flag) to 700 bp. For the RNA‐seq data of the parental taxa, we did not include read pair information (as read pairs could span large and unpredictable intron junctions; i.e., we input each with the ‐U flag). We estimated mapped read coverage with QualiMap v2.2.1 (García‐Alcalde et al., 2012).

We obtained mtDNA sequences from the hybrid reads by focusing on the region typically sequenced by the avian *cytochrome oxidase I* (*COI*) primers (CO1BirdF1 and CO1BirdR2; Herbert et al., 2004) that span positions 6656 and 7405 of mtDNA sequence MH700631. We extracted the 749 bp consensus sequence for the reads from the putative hybrid from these positions using Geneious v11.0.3. We used BLAST‐n to search the NCBI database to identify the most likely maternal parent species.

**FIGURE 3:**
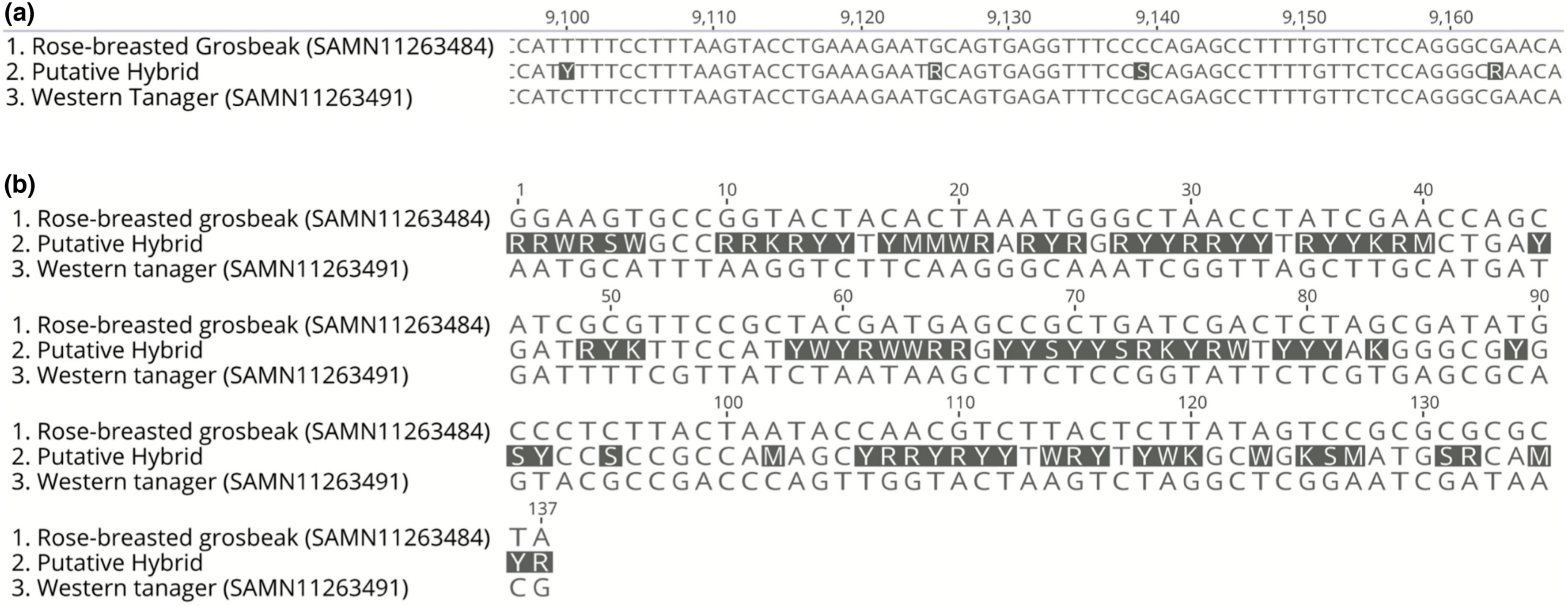
Sequence variation showing heterozygosity and intermediate genotypes of the hybrid Scarlet Tanager × Rose‐breasted Grosbeak compared to parental genera, including a western tanager (MSB:Bird:47847) as the *Piranga* representative. (a) Illustrates a small (50 bp) region of the *LNPEP* gene with multiple heterozygous sites (represented by ambiguous nucleotides). (b) The same *LNPEP* gene, but condensed to those 137 sites where the parental genera differed in non‐ambiguous nucleotides, and where there were no completely ambiguous (“N”) nucleotides for any of the three sequences.

We compared the sequence of hybrid reads to the parental species from an arbitrary portion of the *C. cardinalis* genome that was from a large scaffold with sufficient coverage from all three species (scaffold JACDOX010000102, between 30,488 and 40,944 bp). We extracted the sequence using the “mpileup” command of Samtools (Li et al., 2009) and compared the sequences in Geneious v11.0.3. This region aligns with the *leucyl/cystinyl aminopeptidase* (*LNPEP*) gene in the *Ficedula albicollis* (FicAlb 1.5; GenBank accession # GCA_000247815.2) genome assembly (Ellegren et al., 2012). The intermediacy of the hybrid was overwhelmingly supported by all genomic regions investigated, and thus we report the results of only this region to illustrate the hybridization patterns (Figure 3).

We also quantified global genome‐wide heterozygosity of the putative hybrid's genome using genotype likelihoods in ANGSD (v0.934; Korneliussen et al., 2014) with the “‐dosaf 1” flag to generate the site frequency spectrum. We then used this information to estimate the fraction of the genome with heterozygous sites. To provide context for these heterozygosity values, we compared the heterozygosity measure from the putative hybrid to the same estimates (from an identical sequencing approach) from a recently published dataset of genomes from 156 individuals across 34 species of *Setophaga* and 2 species of *Vermivora* warbler (Parulidae; NCBI SRA# PRJNA630247; Baiz et al., 2021). We used this warbler dataset as their family, Parulidae, is closely aligned to Cardinalidae (Barker et al., 2015) and similar sequencing was not available for other cardinalids.

## 3 RESULTS

In the hand, we confirmed that the putative hybrid was an 1‐year‐old male based on its wing molt limits (Mulvihill, 1993) and cloacal protuberance. Morphometric comparisons (Table 1) show the hybrid intermediate in size between the smaller scarlet tanager and the heavier, more robust rose‐breasted grosbeak. The bill and tail were particularly long.

Qualitative spectrographic analysis of two of the vocalization recordings illustrated that the individual's song and call were comparable to those typical of scarlet tanagers, but not of rose‐breasted grosbeaks. In two recordings, the bird sang 25 bouts of song and 1 partial song/call. The hybrid's song had a “burry” tone produced by rapid frequency modulation; a quality typical of scarlet tanager but not rose‐breasted grosbeak (Figure 2). This quality is visible as a wide bandwidth sound on a low‐resolution spectrogram; in contrast, the tonal sound of a rose‐breasted grosbeak appears as a thin line. On high‐resolution spectrograms, this quality can be resolved as a rapidly oscillating thin tone (Figure S1). Additionally, in the middle of one song, the putative hybrid produced a “chick‐burr” vocalization, highly similar to the same vocalization made by scarlet tanagers.

Quantitative analysis of the recordings confirmed that the individual's song was within the range of scarlet tanager, but largely dissimilar to that of rose‐breasted grosbeak. The number of syllables within the songs varied between 4 and 6 (mean ± SD: 4.96 ± 0.68; *n* = 25), which is within the typical range of scarlet tanager but fewer than the average ~10 syllables of rose‐breasted grosbeak (Table 2). The duration of the song varied from 1.28 s to 2.30 s (mean ± SD: 1.79 ± 0.25; *n* = 25), which was within the typical range for scarlet tanager but shorter than that of rose‐breasted grosbeak (Table 2). The full frequency range of the hybrid song was 1.2 kHz–5.9 kHz. The reported typical ranges for scarlet tanager (2.2–5.5 kHz) and rose‐breasted grosbeak (1.5‐5 kHz) both fall within this range, making frequency range an uninformative feature for this identification.

Sequencing resulted in 59,956,127 reads for the hybrid—including collapsed paired reads—95.9% of which mapped to the 1.04Gbp *C. cardinalis* genome. This produced an average coverage of 8.2X across the genome, with over 95% of the genome sampled by at least one read.

BLAST results from 749 bp of the *COI* region for the hybrid returned a top hit of a rose‐breasted grosbeak, *Pheucticus ludovicianus* voucher 1B‐2600 (99.9% identity; GenBank Accession #EU525468.1), confirming the maternal parent, with the next four hits all *Pheucticus ludovicianus* with identities >99%, and subsequent hits <96% of other *Pheucticus* species.

In the 10,392 bp LNPEP exon region, there were 137 sites where the two putative parental genera (*Pheucticus* and *Piranga)* differed in non‐ambiguous nucleotides and where there were no completely ambiguous (“N”) nucleotides for any of the three sequences. For 85 (62%) of these 137 sites, the putative hybrid was heterozygous, with intermediate genotypes between the two parental genera represented by the concordant ambiguous nucleotides (Figure 3). At 24 (18%) of the sites, the hybrid matched the base found in *Pheucticus ludovicianus*, and at 26 of the sites (19%), the hybrid matched *Piranga ludoviciana*. At two sites, the ambiguous nucleotide did not match the base in one of the two parental taxa.

The hybrid's genome was exceptionally heterozygous. The mean heterozygosity estimate for 156 warbler individuals was 0.007, with a maximum value 0.0163. The putative Scarlet Tanager x Rose‐Breasted Grosbeak hybrid had a heterozygosity value of 0.037, over 5 standard deviations above the highest value from the warbler dataset.

## 4 DISCUSSION

The combination of evidence—visual, bioacoustic, and genetic—confirms that the parents of the described individual were a rose‐breasted grosbeak *Pheucticus ludovicianus* (female parent) and a scarlet tanager Piranga olivacea (male parent). While these two species breed sympatrically across much of eastern North America, they exhibit somewhat different habitat preferences: scarlet tanagers typically prefer unfragmented, mature forest, while rose‐breasted grosbeaks often will occupy second growth including forest with a relatively open canopy, although they will utilize adjacent edges or disturbed areas (Mowbray, 2020; Wyatt & Francis, 2020). The two species are phenotypically highly divergent and have likely not shared a common ancestor in >10 million years (Barker et al., 2015).

Our qualitative and quantitative analyses of the song showed that the vocalizations of this individual were highly similar to those of scarlet tanager and largely dissimilar to those of rose‐breasted grosbeak. This individual's rapidly frequency‐modulated song and “chick‐burr” call were qualitatively very similar to the scarlet tanager's song and call, whereas rose‐breasted grosbeaks do not produce rapidly frequency‐modulated songs or “chick‐burr” calls. In addition, the average number of syllables per song and the song duration were within range of the scarlet tanager song but exceeded that of the rose‐breasted grosbeak song.

In addition to the analysis described above, we also used the “Merlin” sound identification mobile application from the Cornell Lab of Ornithology to evaluate our identification. This algorithm was trained on curated song recordings deposited in the Macaulay Library and can identify over 400 species by vocalization in North America. When playing the hybrid's song recording for the software, the program invariably identified it as a scarlet tanager, in line with our more detailed assessment of song characteristics described above. We note, however, that the trained model, architecture, and underlying data of the Merlin Sound ID feature have not been published, the classifier accuracy has not been described in the literature, and uncertainty of individual classifications is unreported, preventing more detailed comment on the context and implications of this result.

Shy (1984) found that scarlet tanagers lack regional dialects, suggesting that this species learns its song in its first breeding season instead of at its natal site. The similarity between the syllables of this bird's song and that of a counter‐singing scarlet tanager suggests that it may have learned its song from its paternal parent or nearby neighbors at this breeding location. Hand‐reared rose‐breasted grosbeaks are unable to sing correctly, suggesting a critical developmental period in this species (Dunham, 1966) but it is unknown how the singing that the bird is exposed to in this critical period correlates with the song ultimately learned by the individual.

**FIGURE 4:**
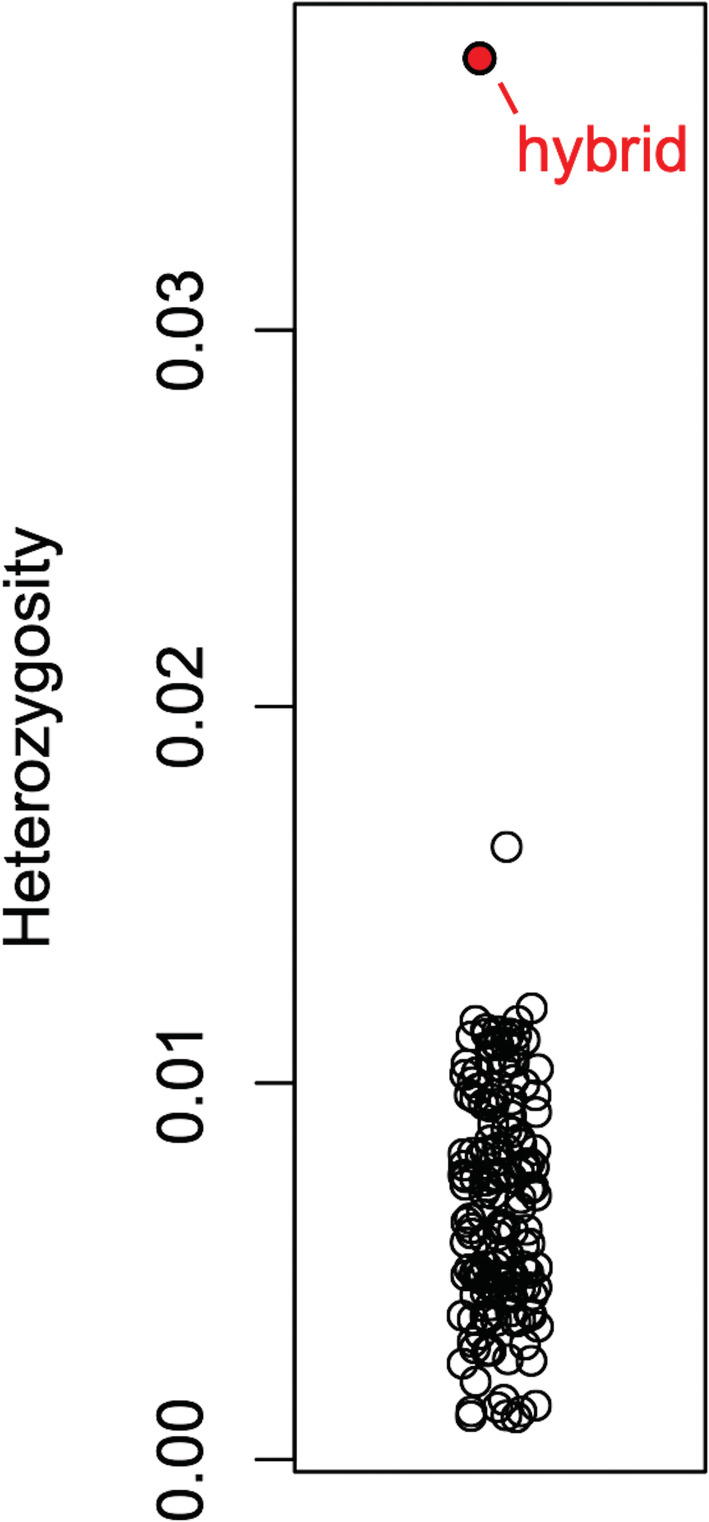
Genome‐wide heterozygosity estimate for 156 wood warblers (family Parulidae; open circles) from two genera (*Setophaga* and *Vermivora*) published previously (Baiz et al., 2021), and the putative Rose‐Breasted Grosbeak × Scarlet Tanager hybrid sequenced here (red filled point).

The genome of the hybrid was exceptionally heterozygous (Figures 3 and 4)—as is expected from an F1 hybrid with highly divergent parents—with a heterozygous base every 100–150 bp. This is also a likely underestimate. First, given that the parental genera were represented by RNA sequence data, the only regions we analyzed in depth here were coding regions, and these regions are constrained by stronger purifying selection than non‐coding sequences (Ward & Kellis, 2012). Second, accurately calling heterozygous sites requires high coverage (Song et al., 2016); thus, we presume that many of the sites that differed between the parental genera but where the hybrid had one or the other genotype (i.e., was not heterozygous), might actually be heterozygous in the hybrid, but we lack the coverage depth to decisively call a heterozygous genotype. The fact that the sites where the hybrid had one or the other parental genotype occur in nearly equal frequencies (24 vs. 26 sites of 137) supports this interpretation.

We also note that while our comparison dataset of low‐coverage warbler genomes did not explicitly include any known hybrids, the level of heterozygosity appeared even higher than in other avian hybrid zones analyzed with genomic data. For example, between myrtle (*Setophaga coronata coronata*) and Audubon's (*S. c. auduboni*) warblers, Toews, Lovette, et al. (2018) used ddRAD sequencing to analyze 19,709 variable SNP loci. Importantly, of those SNPs, only 87 were divergent enough (i.e., *F*_ST_ > 0.7) to calculate inter‐specific heterozygosity estimates in hybrids. In other words, as expected, based on their last common ancestor, the genomes of the two parental species here are highly divergent and are manifested >1% of the hybrid's genome being heterozygous.

The hybrid described here has parental species on the upper end of divergence times in natural hybrids (i.e., not in domesticated species or produced in artificial settings) described with sufficient evidence. There have been several *Anser* x *Branta* hybrids (e.g., *Anser anser* x *Branta canadensis* or *Anser albifrons* x *Branta canadensis*), which diverged approximately 12 mya (Sun et al., 2017). A hybrid between *Aglaiocercus kingii* x *Metallura tyrianthina*, known as the “Rogitama hummingbird,” was originally described by Stiles and Cortés‐Herrera (2015), and further analysis was later provided by Pérez‐Emán et al. (2017); these species diverged approximately 10 mya (McGuire et al., 2014). *Himantopus mexicanus* x *Recurvirostra americana* diverged approximately 30 mya and hybrids possibly occur in the wild; however, the only records of these hybrids come from captive birds (i.e., Principe Jr, 1977). Finally, a putative *Icteria virens* x oriole *sp*. hybrid was recently identified, where the parental species would have diverged approximately 10 mya (Oliveros et al., 2019), but molecular confirmation of this hybrid is still in progress (A. Brelsford pers. comm.).

It would be ideal to put the described hybrid Rose‐Breasted Grosbeak x Scarlet Tanager into context by comparing its estimated heterozygosity to the heterozygosity values of other highly divergent hybrid taxa; however, few estimates from such taxa exist given data limitations and difficulties of obtaining genetic material from wild hybrids. We recommend that future researchers consider heterozygosity estimation a priority to facilitate comparisons that may unveil evolutionary patterns.

An important caveat to our work is that while we were able to determine genetic parentage with very high confidence, our evidence was not 100% confirmed, as we were only able to include nuclear data from a congener for one of the parental taxa. We could have achieved near perfect certainty in confirming parental taxa by including additional sequencing of both parental species. However, the strength of morphological, bioacoustic, and genetic evidence supports that the parents of this hybrid were a rose‐breasted grosbeak and a scarlet tanager, and additional sequencing would be unlikely to yield new insight.

Documentation and identification of this hybrid support the utility of low‐coverage whole‐genome sequencing, particularly when combined with diverse data archives and bioacoustic information, as a straightforward method to assign ancestry for putative hybrid individuals. More generally, the observation that this individual—between such highly divergent parental taxa—lived until adulthood and behaved like a typical territorial passerine, serves as another example of the survival capacity of birds with exceptionally heterozygous genomes. We note, however, that we could not verify reproduction by this individual hybrid, and a careful search for the bird on territory in 2021 was unsuccessful.

## AUTHOR CONTRIBUTIONS

**David P. L. Toews:** Conceptualization (equal); formal analysis (lead); funding acquisition (lead); investigation (lead); writing – original draft (lead); writing – review and editing (lead). **Tessa A. Rhinehart:** Formal analysis (supporting); methodology (supporting); visualization (equal); writing – original draft (supporting); writing – review and editing (supporting). **Robert Mulvihill:** Conceptualization (equal); investigation (lead); writing – original draft (supporting); writing – review and editing (supporting). **Spencer Galen:** Formal analysis (supporting); methodology (supporting); writing – review and editing (supporting). **Stephen M. Gosser:** Conceptualization (equal); formal analysis (supporting); writing – review and editing (supporting). **Tom Johnson:** Conceptualization (supporting); writing – review and editing (supporting). **Jessie L. Williamson:** Formal analysis (supporting); methodology (supporting); writing – review and editing (supporting). **Andrew W. Wood:** Formal analysis (supporting); writing – review and editing (supporting). **Steven C. Latta:** Conceptualization (equal); investigation (lead); writing – original draft (supporting); writing – review and editing (supporting).

## CONFLICT OF INTEREST

The authors declare no conflict of interest.

## Supporting information


Figure S1

## Data Availability

Genome sequence of putative hybrid: NCBI SRA: Bioproject #PRJNA861242. Recording: MacAulay Library of Natural Sounds: Asset ML462228091, ML462228311, ML462228571, ML462231831. Recording annotation files: https://doi.org/10.5061/dryad.wm37pvmqs.
