# Supplementary material for: Genetic confirmation of a hybrid between two highly divergent cardinalid species: A rose‐breasted grosbeak (Pheucticus ludovicianus) and a scarlet tanager (Piranga olivacea)
Source: Ecol Evol. 2022 Aug 1;12(8):e9152. doi: 10.1002/ece3.9152 (PMC9343856; doi:10.1002/ece3.9152)
Supplement: Supplementary file 1 — Figure S1 [file ECE3-12-e9152-s001.pdf]

Electronic Supplementary Material for:

Genetic confirmation of a hybrid between two highly divergent cardinalid species: a Rose-breasted Grosbeak (*Pheucticus ludovicianus)* and a Scarlet Tanager *(Piranga olivacea)*

David P. L. Toews[1], Tessa A. Rhinehard[2], Robert Mulvihill[3], Spencer Galen[4], Stephen M. Gosser[5], Tom Johnson[6], Jessie Williamson[7], Andrew W. Wood[1], and Steven C. Latta[3]

1) Department of Biology, 619 Mueller Laboratory, Pennsylvania State University, University Park, Pennsylvania 16802, toews@psu.edu
2) Department of Biological Sciences, University of Pittsburgh, Pittsburgh, PA 15260 tessa.rhinehart@pitt.edu
3) Department of Conservation and Field Research, National Aviary, Pittsburgh, PA 15212, robert.Mulvihill@aviary.org
4) Biology Department, University of Scranton, Scranton, PA 18510, spgalen@gmail.com
5) Pittsburgh, PA, sgosser72@gmail.com
6) The Academy of Natural Sciences of Drexel University, Philadelphia, PA, tbj4@cornell.edu
7) Department of Biology and Museum of Southwestern Biology, University of New Mexico, Albuquerque, NM 87131

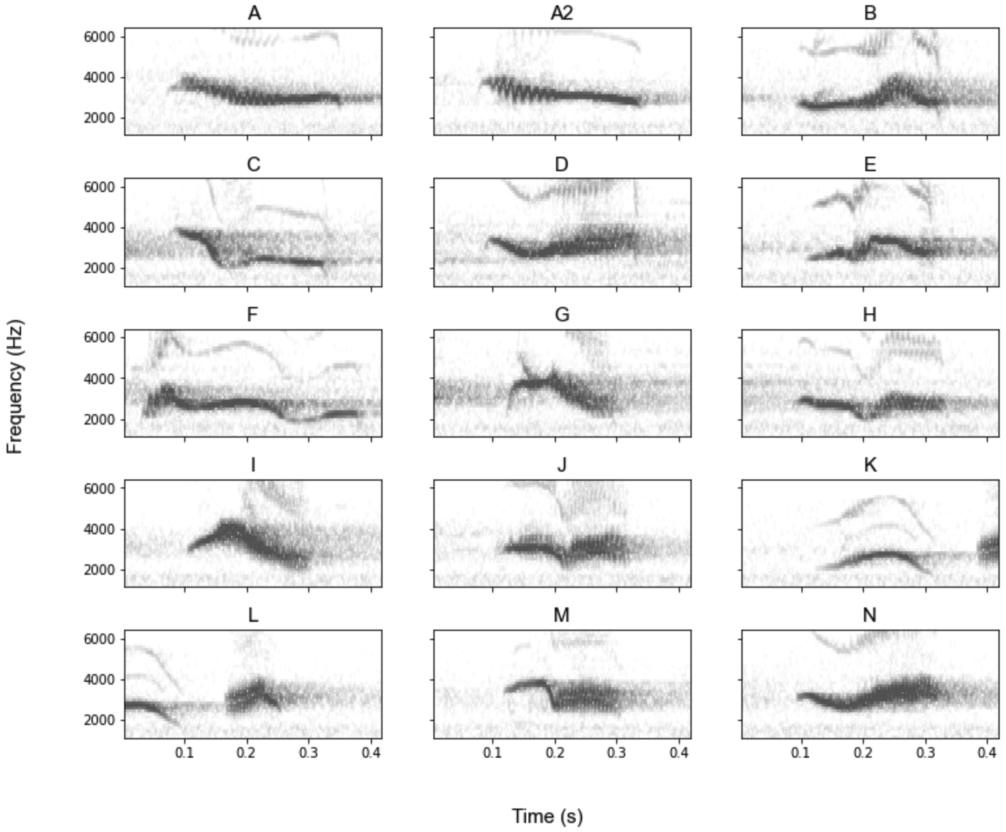

Fig S1. Syllable annotations from recordings of the putative hybrid.
